# Supplementary material for: Y chromosome introgression between deeply divergent primate species
Source: Nat Commun. 2024 Nov 29;15:10398. doi: 10.1038/s41467-024-54719-8 (PMC11607401; doi:10.1038/s41467-024-54719-8)
Supplement: Supplementary file 1 — Supplementary Information [file 41467_2024_54719_MOESM1_ESM.pdf]

Y chromosome introgression between deeply divergent primate species

Axel Jensen, Emma R. Horton, Junior Amboko, Stacy-Anne Parke, John A. Hart, Anthony J. Tosi, Katerina Guschanski, Kate M. Detwiler

Supplementary Figures 1-15

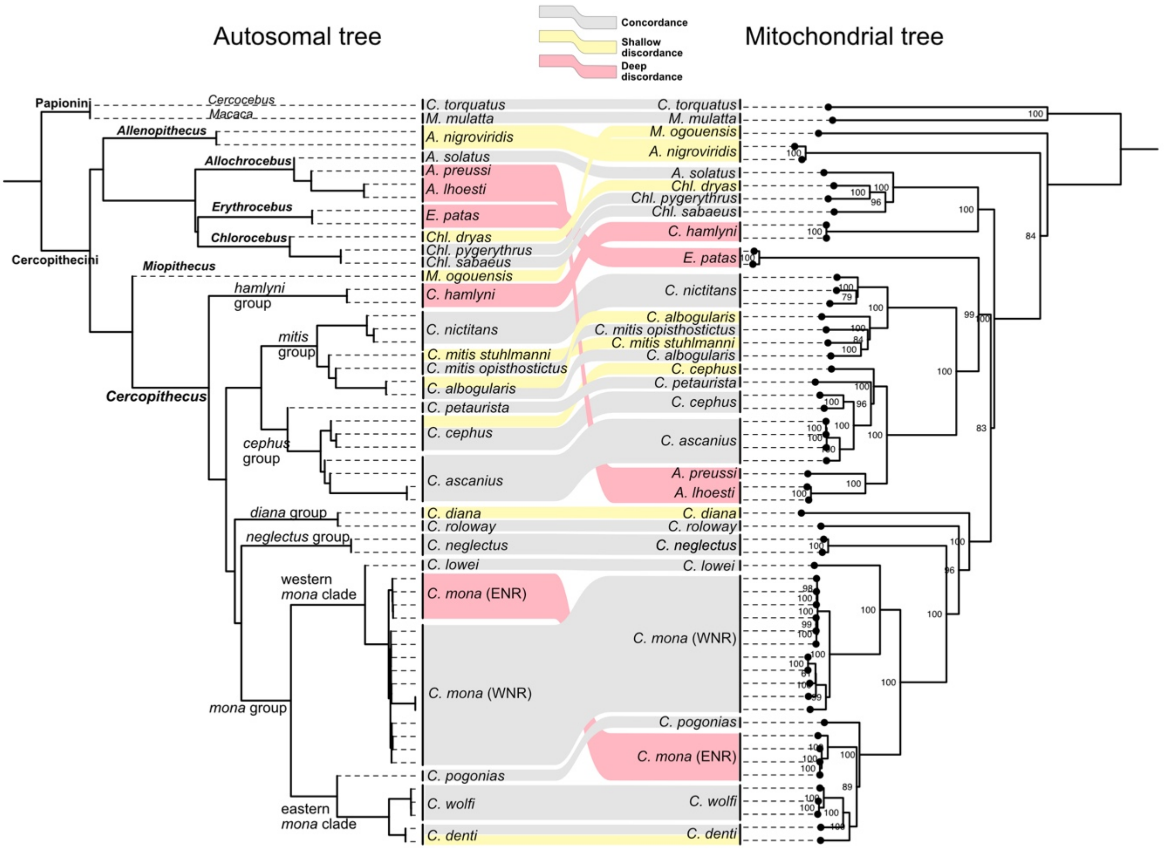

Supplementary Figure 1. Mito-nuclear conflicts. Concordant phylogenetic positions between the autosomal and the mtDNA trees are shown by gray connections. Shallow discordances that can be the result of ILS or introgression are shown in yellow, whereas deep discordances, which more likely result from introgression, are shown in red. Source data are provided as a Source Data file.

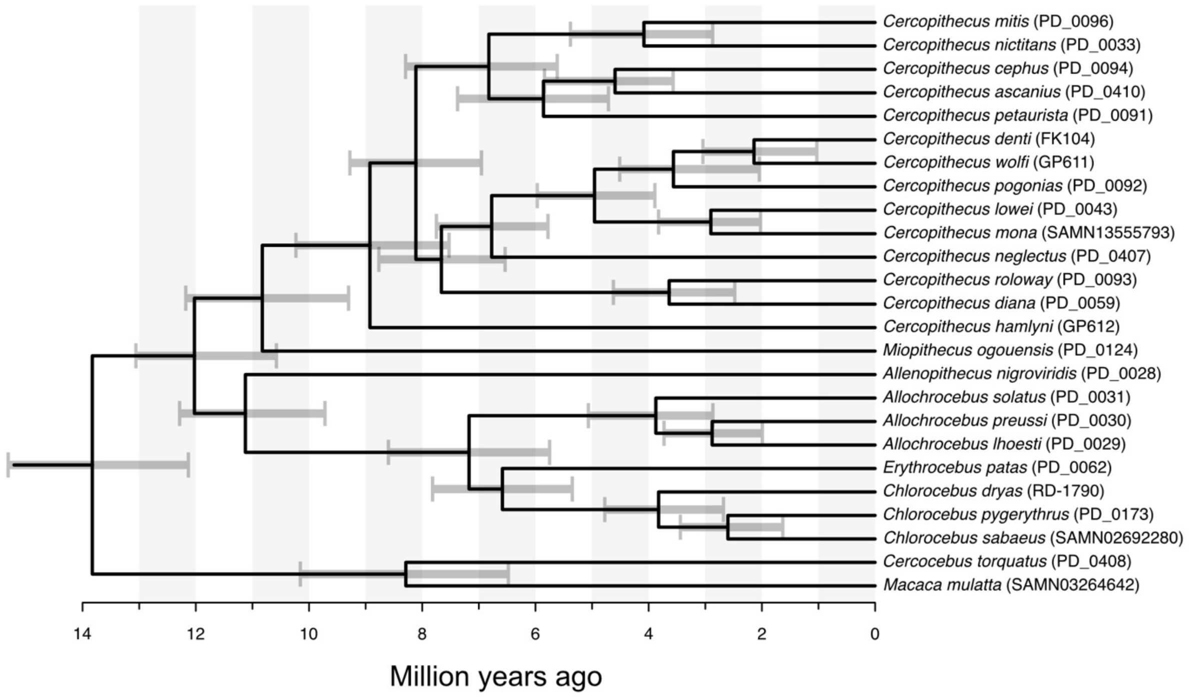

Supplementary Figure 2. Autosomal divergence date estimates for all included species using MCMCTree. Divergence dates were estimated using a single sample per species (ID in parenthesis, table S1). Gray bars correspond to 95% HPD credible intervals. Source data are provided as a Source Data file.

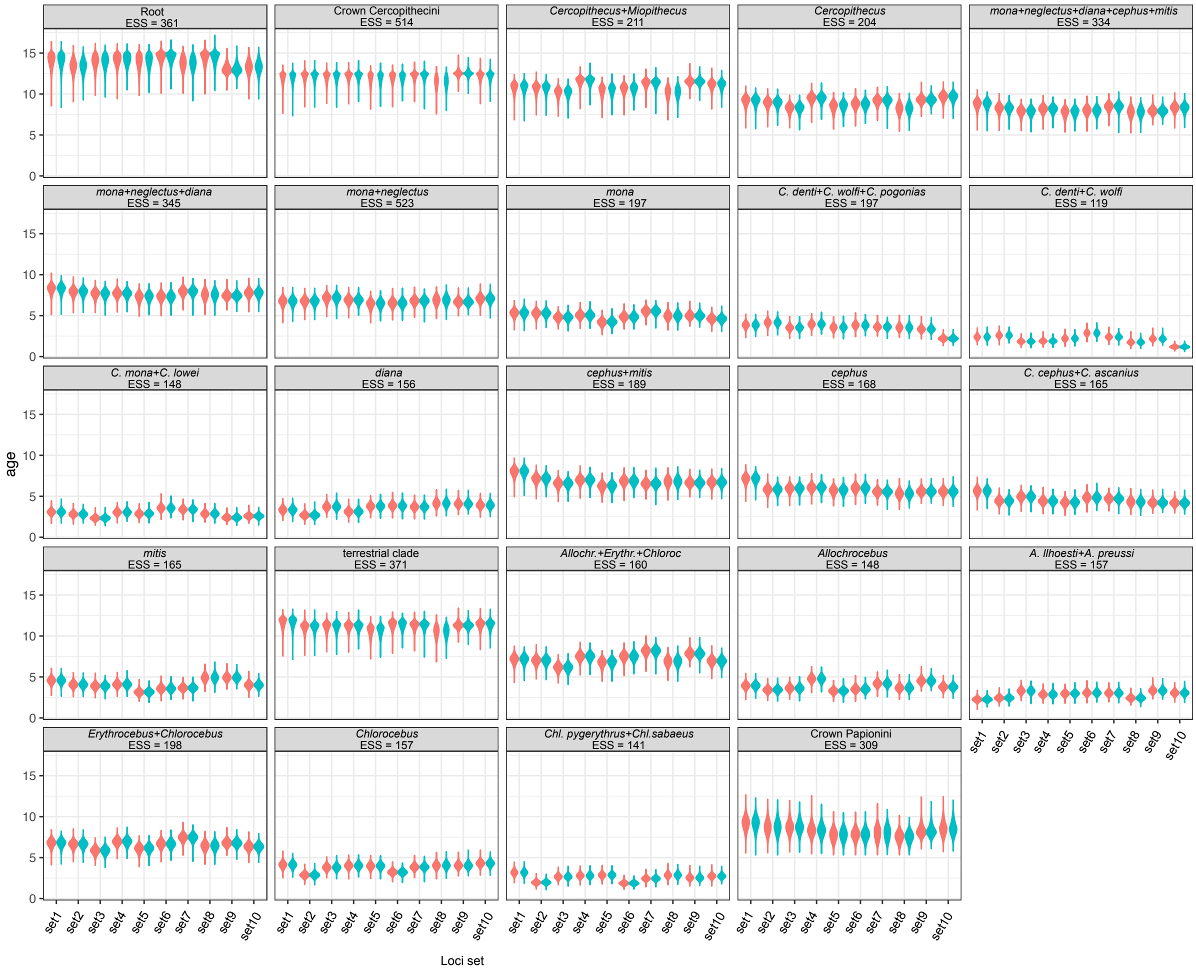

Supplementary Figure 3. Autosomal date estimate distribution/MCMCTree convergence. Age distributions among all autosomal MCMCTree runs shown as violin plots. The analysis was run on ten independent loci (set 1-10), and each set was analyzed in two independent runs (the two runs for each locus are color coded red and green). Age on the Y-axis given in million years. The nodes to which the age estimates apply are given above each panel, together with the obtained Effective Sample Size (ESS). Source data are provided as a Source Data file.

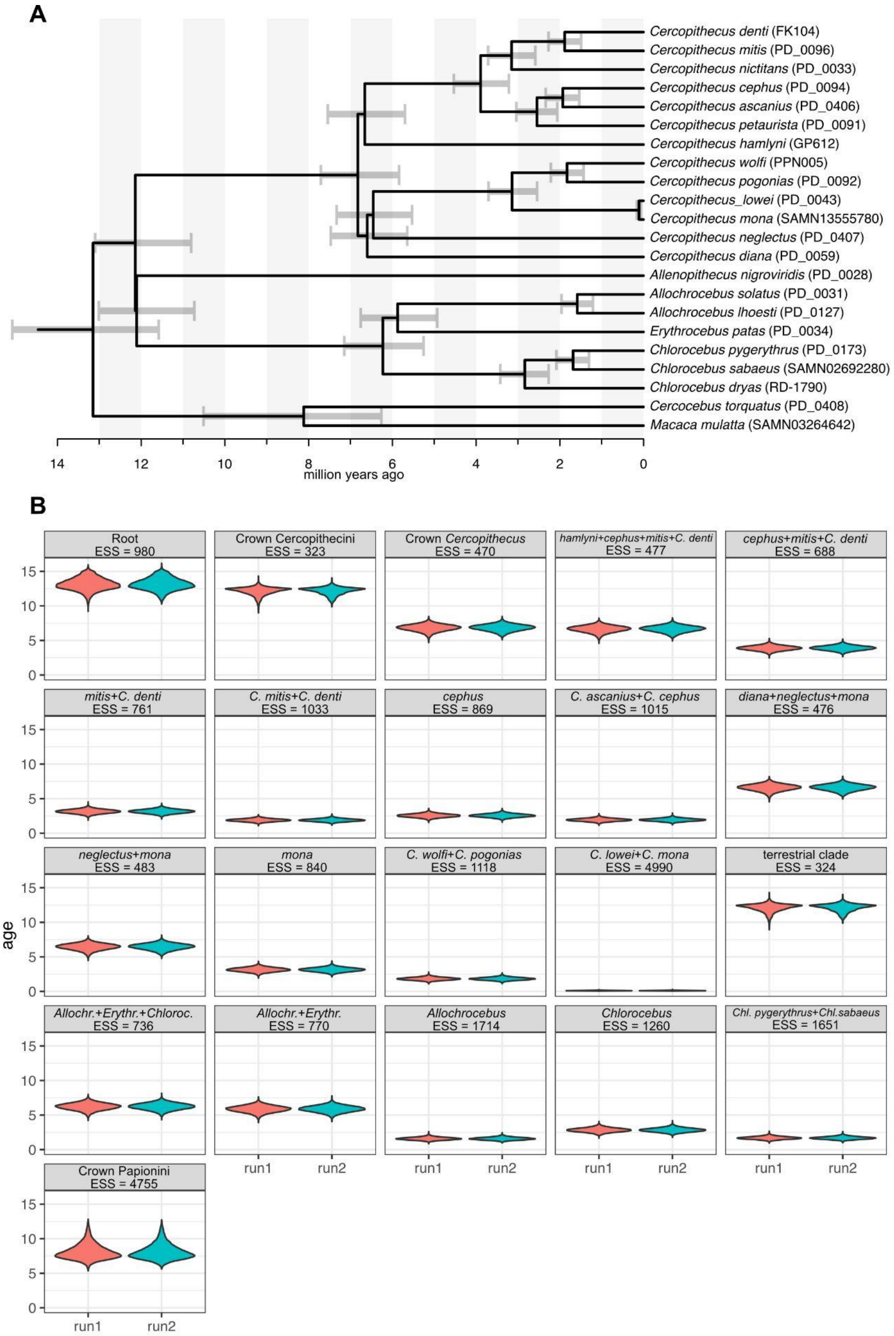

Supplementary Figure 4. A) Y-chromosomal divergence date estimates for all species in the data set. B) Age distributions among all Y-chromosomal MCMCTree runs shown as violin plots. The analysis was run on a Y chromosome alignment (see Methods), analyzed in two independent runs. Age on the Y-axis given in million years. The nodes to which the age estimates apply are given above each panel, together with the obtained Effective Sample Size (ESS). Source data are provided as a Source Data file.

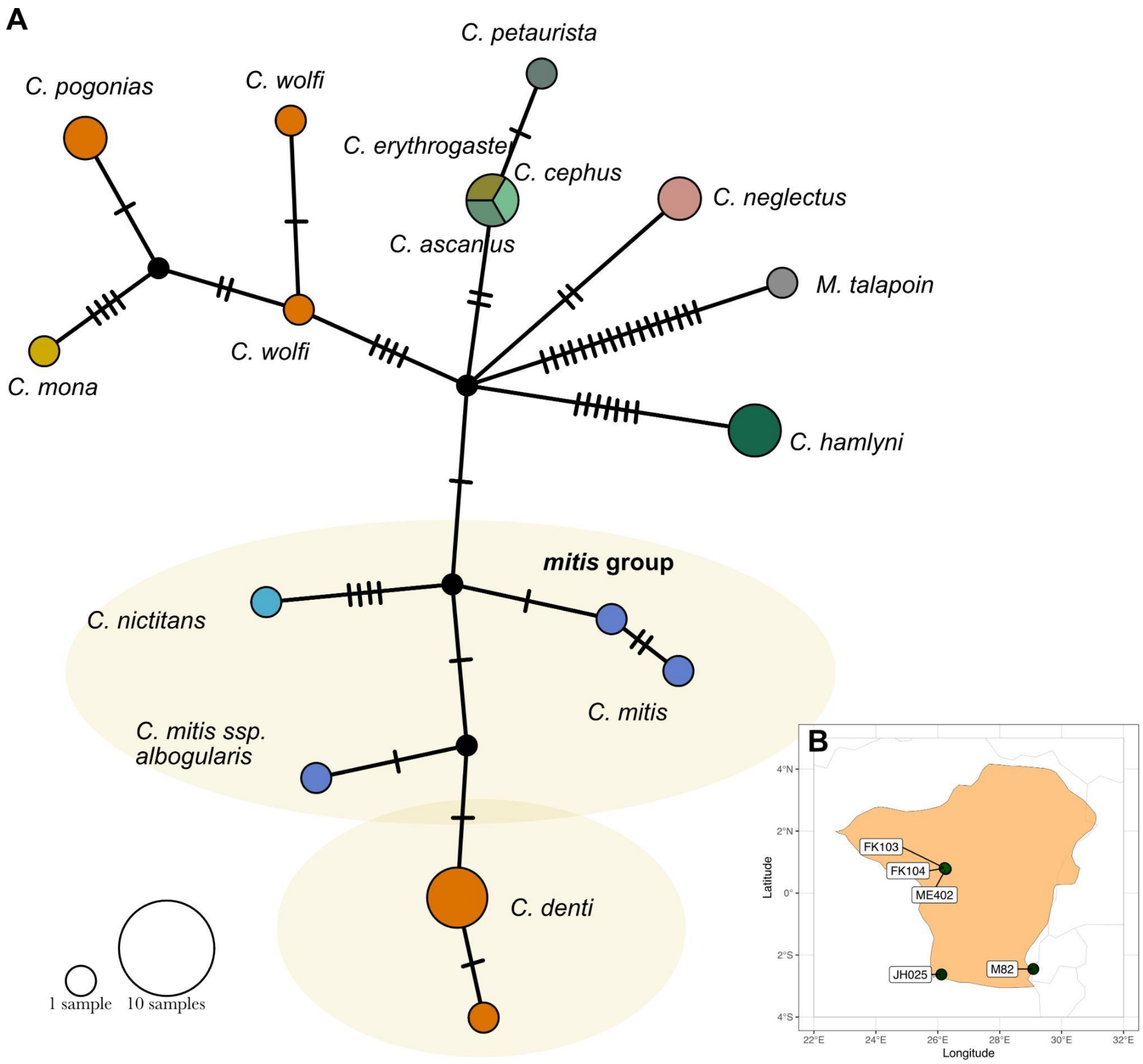

Supplementary Figure 5. A) Median joining network based on 898 bp from the Y-linked TSPY locus. The network includes five *C. denti* individuals, sampled across the distribution range, and shows that the introgressed *mitis*-like Y-chromosome is present in all study samples. B) Map showing the distribution range of *C. denti* (orange) with points showing the sampling locations of the sequenced individuals. Source data are provided as a Source Data file.

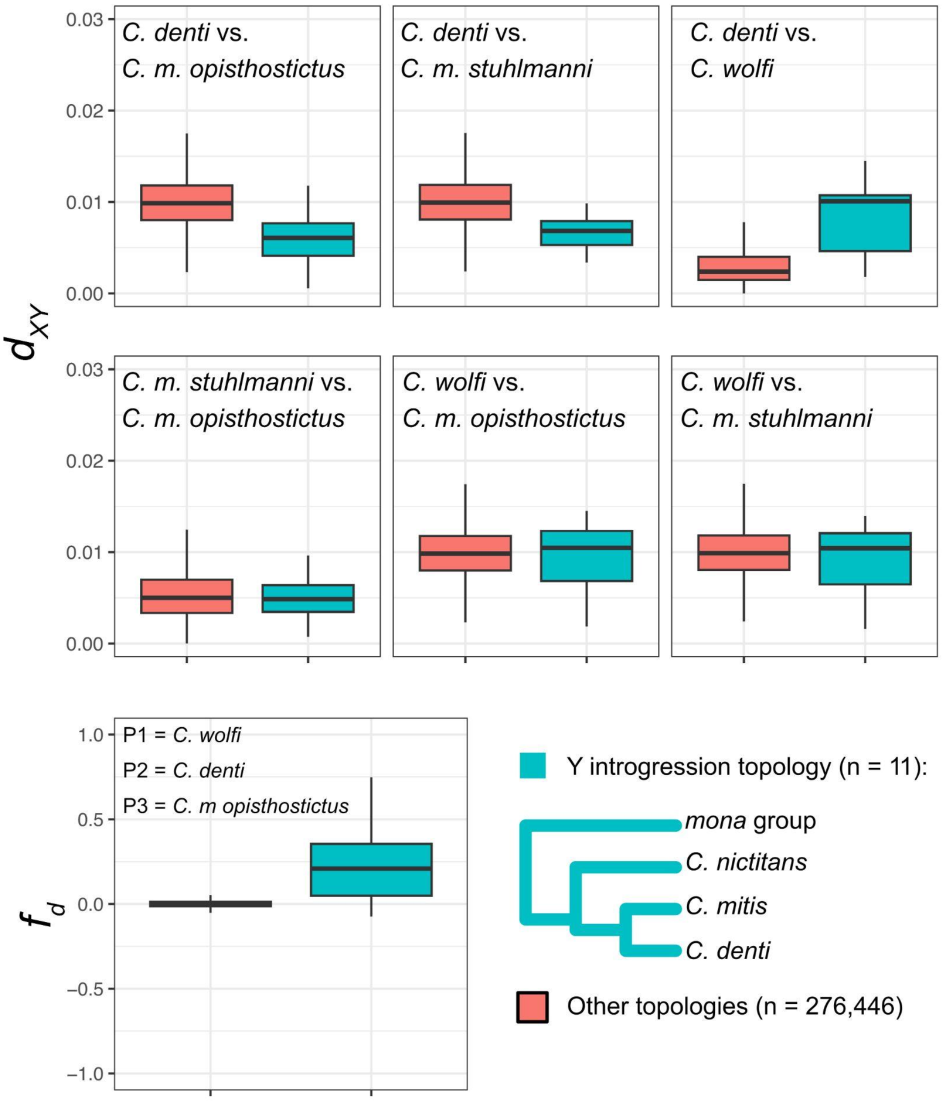

Supplementary Figure 6. Patterns of pairwise autosomal sequence divergence (d_{XY}) in genomic windows of different topological relationships. In blue are windows with a sister relationship between *C. denti* and *C. mitis* inside a monophyletic *mitis* group clade, to the exclusion of a monophyletic *mona* group clade. This topology corresponds to the Y-chromosomal relationship. In red are all other topologies. d_{XY} values are shown for different combinations of taxa. The bottom panel demonstrates that blue windows show elevated f_d, consistent with them being the result of introgression. Boxplot elements: center line, median; hinges, first/third quartile; whiskers, observations in 1.5x interquartile range. Source data are provided as a Source Data file.

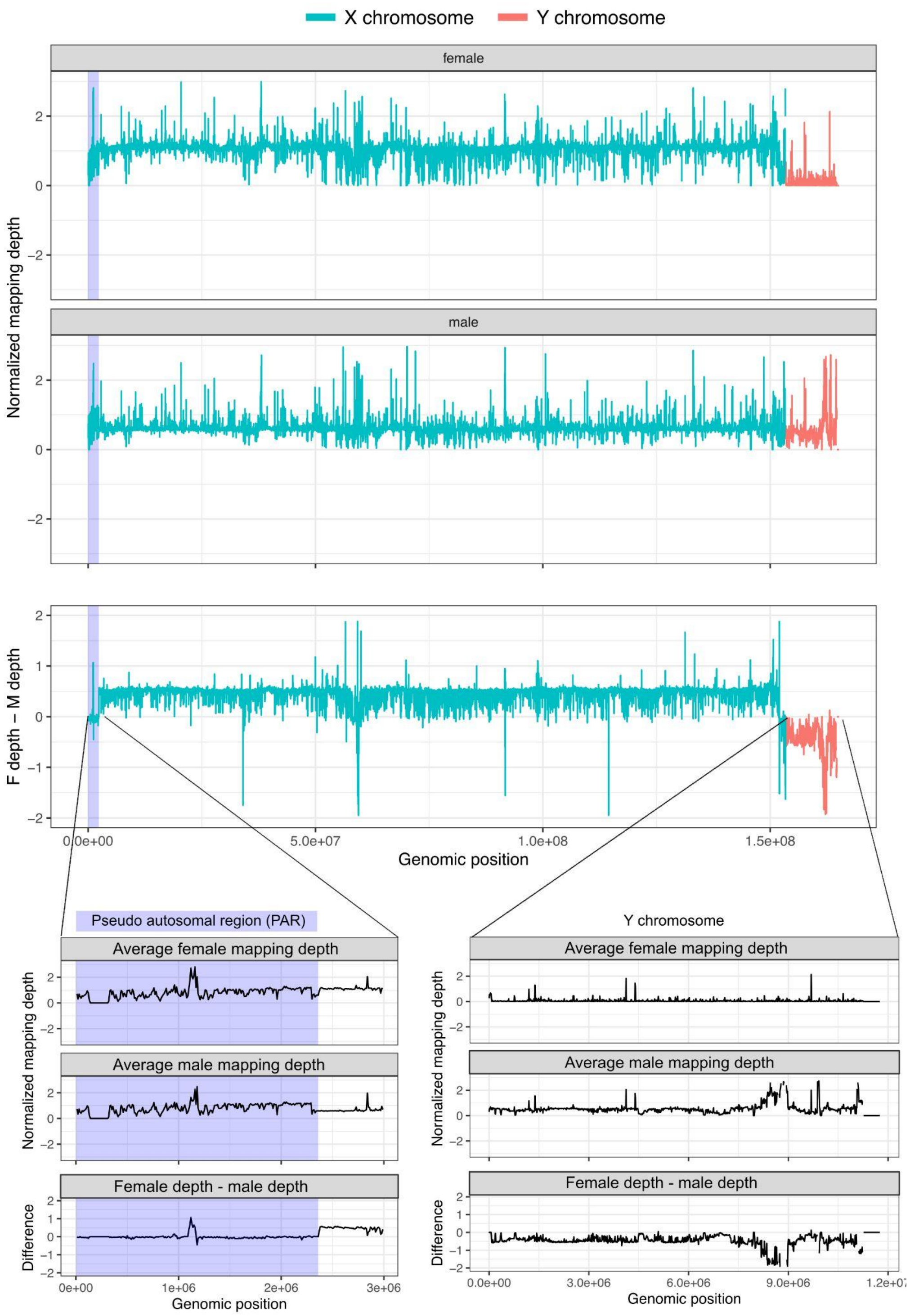

Supplementary Figure 7. Identification of the pseudo-autosomal region (PAR) in the rhesus macaque reference genome based on female vs. male mapping depth. Mapping depth was normalized by the autosomal depth. Since the PAR is present on both X and Y chromosomes, this region is expected to show a normalized depth of ~1 in both females and males. The non-PAR part of the X chromosome is expected to show a depth of ~1 in females and ~0.5 in males, whereas the Y chromosome depth is expected to be 0 in females and ~0.5 in males. Based on this, we identified a PAR region in the first ca. 2.36 Mb of the X chromosome (highlighted in purple), whereas no such region was found on the reference Y chromosome. Source data are provided as a Source Data file.

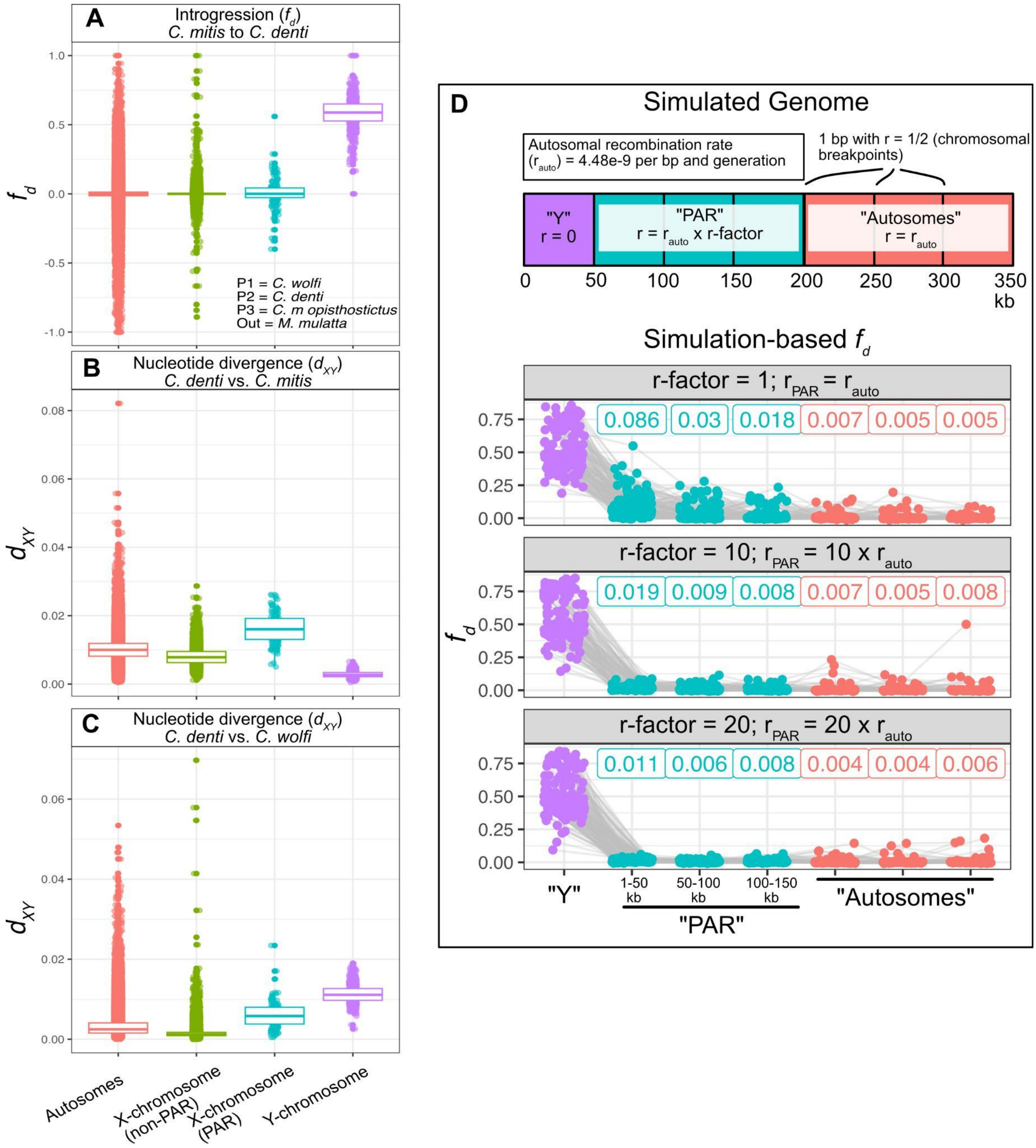

Supplementary Figure 8. Introgression and divergence on the autosomes, X-chromosome, X-linked PAR region, and the Y-chromosome**.** Excess allele sharing (f_d) between *C. denti* and *C. mitis* (A) and nucleotide divergence (d_{XY}) of *C. denti* and *C. mitis opishtostictus* (B), and *C. denti* and *C. wolfi* (C), in regions on the autosomes, X chromosome (PAR and non-PAR) and Y chromosome. Boxplot elements: center line, median; hinges, first/third quartile; whiskers, observations in 1.5x interquartile range. D) Simulations exploring the expected allele sharing (f_d) on the PAR in the event of Y chromosome introgression, with different PAR recombination rates. The “Y” locus was simulated as a non-recombining region of 50 kb, linked to a “PAR” region of 150 kb with either the same (r-factor = 1) or increased (r-factor = 10 and 20) recombination rate relative to the “Autosomes”, which were simulated as 50 kb regions separated by 1 bp with a recombination probability of 0.5, effectively reflecting unlinked loci. f_d was estimated in 50 kb loci along each replicate “genome” simulated (illustrated by gray connectors). Colored labels indicate mean, window-specific *fd* across all replicates. Source data are provided as a Source Data file.

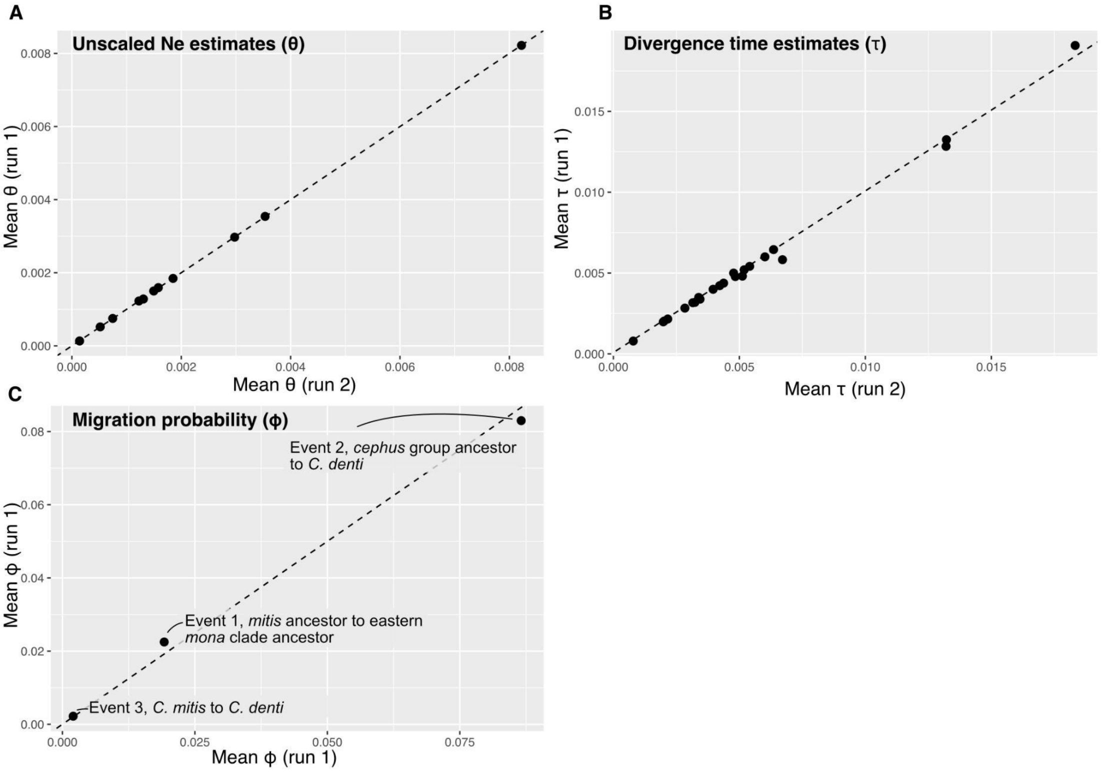

Supplementary Figure 9. BPP convergence between two independent runs of the MSCi model. Comparisons between two independent BPP-MSci runs (Figure 5 in the main text). A) Theta (θ) estimates, where each point represents the estimated unscaled effective population size (Ne) for one branch in the tree. B) Unscaled divergence time estimates, tau (τ), the estimated divergence time for each internal node is represented by one point. C) Migration probability estimates, phi (φ), for the three modeled gene flow events (event 1, 2 and 3 in Figure 5 in the main text). Source data are provided as a Source Data file.

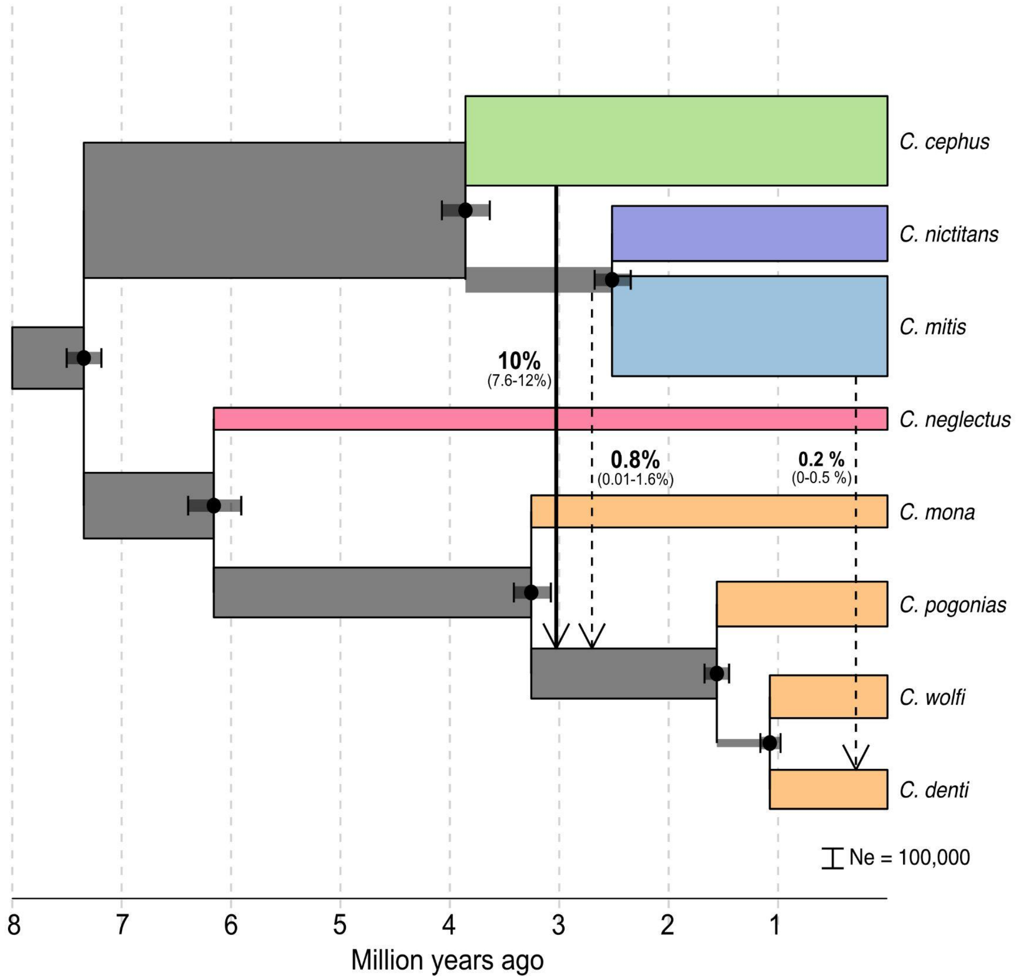

Supplementary Figure 10. Demographic history of focal lineages using BPP and the same model as in main Figure 5, except that the gene flow event from *C. cephus* into the *C. pogonias/wolfi/denti* ancestor precedes that from the *mitis* group ancestor. Arrows show the modeled gene flow events, labelled with the estimated migration proportion and 95 % highest posterior distribution credible interval. Branch lengths and widths were scaled to years and effective population size, respectively, using a generation time of 10 years and a mutation rate of 4.82e-9. Source data are provided as a Source Data file.

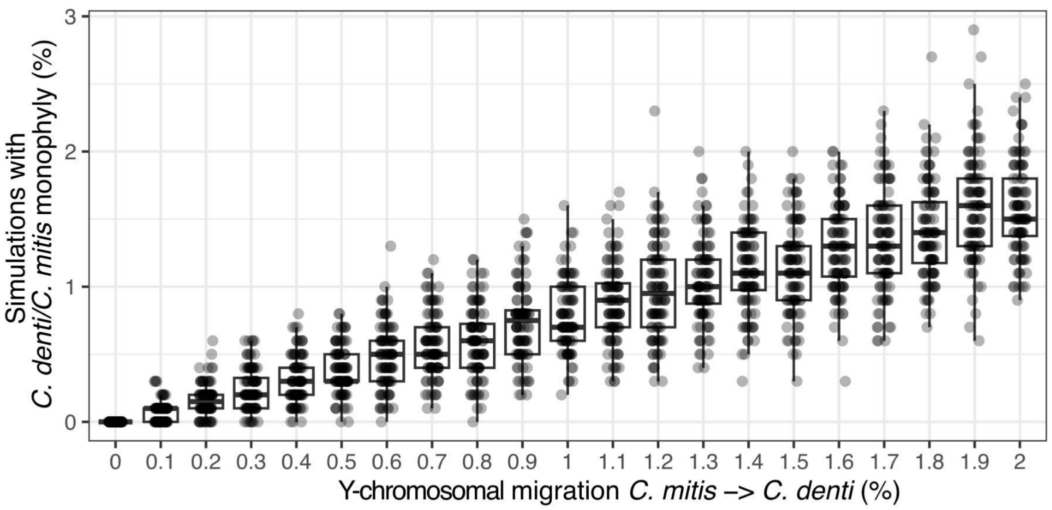

Supplementary Figure 11. Frequencies of a sister relationship between *C. denti* and *C. mitis* on the Y chromosome under different rates of migration (event 3 in Figure 5). All simulations also include migration events from the *mitis* group ancestor and the *cephus* group ancestor into the ancestor of the eastern *mona* clade (2.1 % and 8.5 %, events 1 and 2, respectively, Figure 5). The Y chromosome was simulated as a single, non-recombining locus with effective population sizes of ¼ of the autosomes. We conservatively assumed that all *C. mitis* migrants entering the *C. denti* population were males, leading to an effective Y-chromosomal migration rate twice that of the autosomal migration rate (0-2%, with a stepwise increase of 0.1 %, to retrieve migration proportions directly equivalent to the autosomal migration rates). We then calculated the proportion of simulations in which *C. denti* and *C. mitis* formed a monophyletic Y-chromosomal clade when randomly sampling one individual per species. We ran 100 replicates, comprising 1000 simulations each, for every migration rate. Under strictly male mediated gene flow, an autosomal migration rate of 0.4 % corresponds to a Y-chromosomal migration rate of 0.8 % (dashed line). At this migration rate, *C. denti* and *C. mitis* were monophyletic in ~0.6 % of the simulations. These simulations confirm that ILS alone or in combination with the ancestral gene flow from the *mitis* group ancestor into the *mona* group ancestor (event 1 in Figure 5) is unlikely to have caused the discordant Y-chromosomal tree position of *C. denti*: Without migration from *C. mitis* into *C. denti* (event 3 in Figure 5, Y-chromosomal gene flow of 0 %), *C. denti* and *C. mitis* never formed a monophyletic clade (out of a total of 100,000 simulations). Boxplot elements: center line, median; hinges, first/third quartile; whiskers, 1.5x interquartile range or min/max. Source data are provided as a Source Data file.

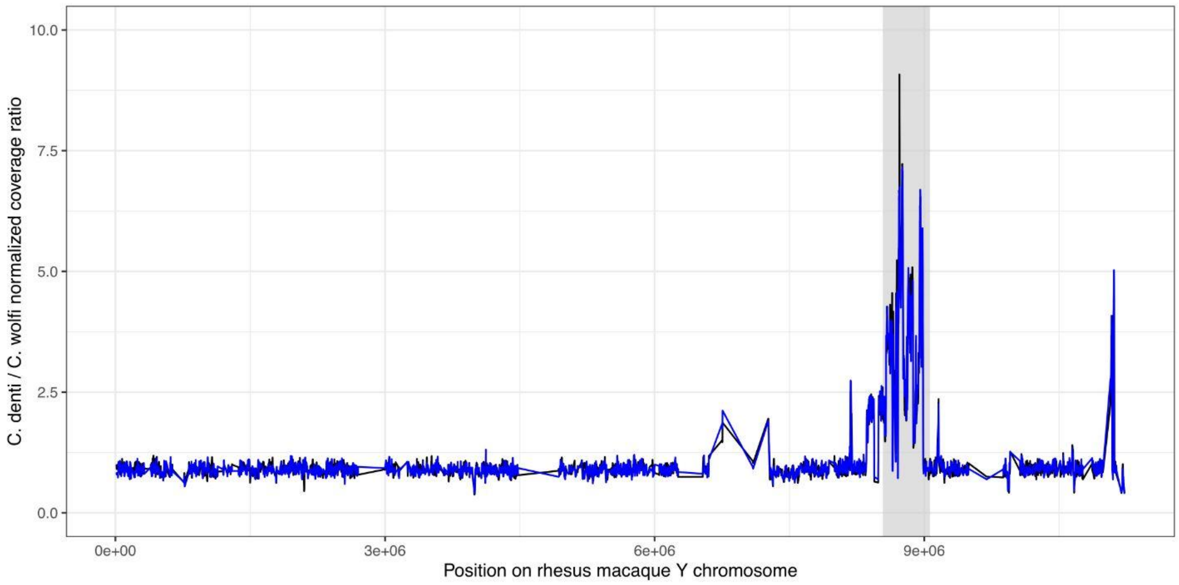

Supplementary Figure 12. Normalized coverage ratio *C. denti* / *C. wolfi* along the rhesus macaque Y-chromosome, calculated separately for both included *C. wolfi* males (blue and black line, respectively). The grey rectangle highlights the region of increased coverage in *C. denti* compared to *C. wolfi*. Source data are provided as a Source Data file.

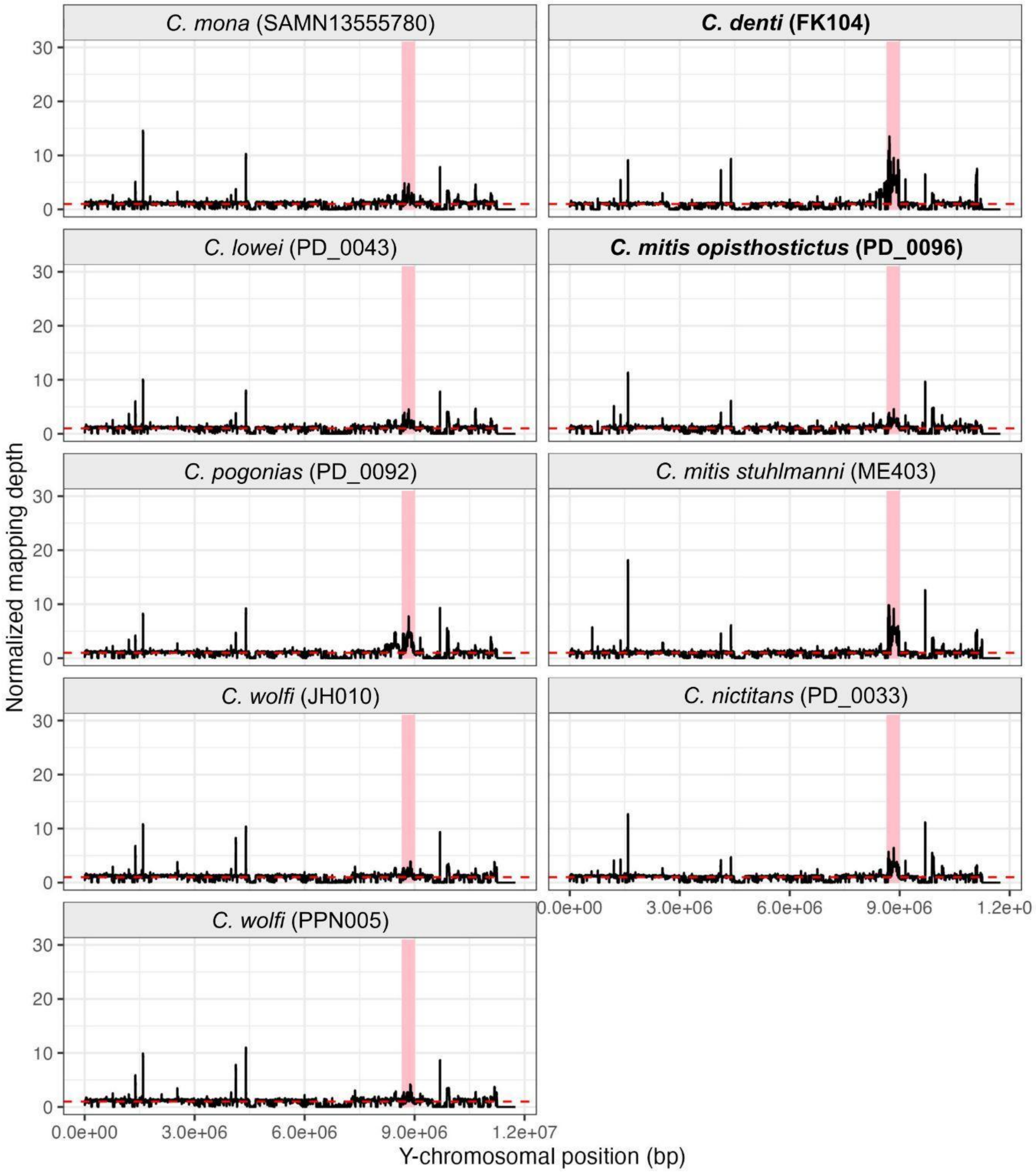

Supplementary Figure 13. Mapping coverage along the macaque Y-chromosome, for a subset of guenon species, normalized by average Y-chromosomal coverage. Left column shows the Y-coverage among *mona* group lineages that retained the ancestral allele, and the right column shows the coverage in *C. denti* and *mitis* group taxa. Horizontal dashed line shows the normalized coverage value of one. The region showing high variation in Y-chromosomal coverage is highlighted by red rectangles. Source data are provided as a Source Data file.

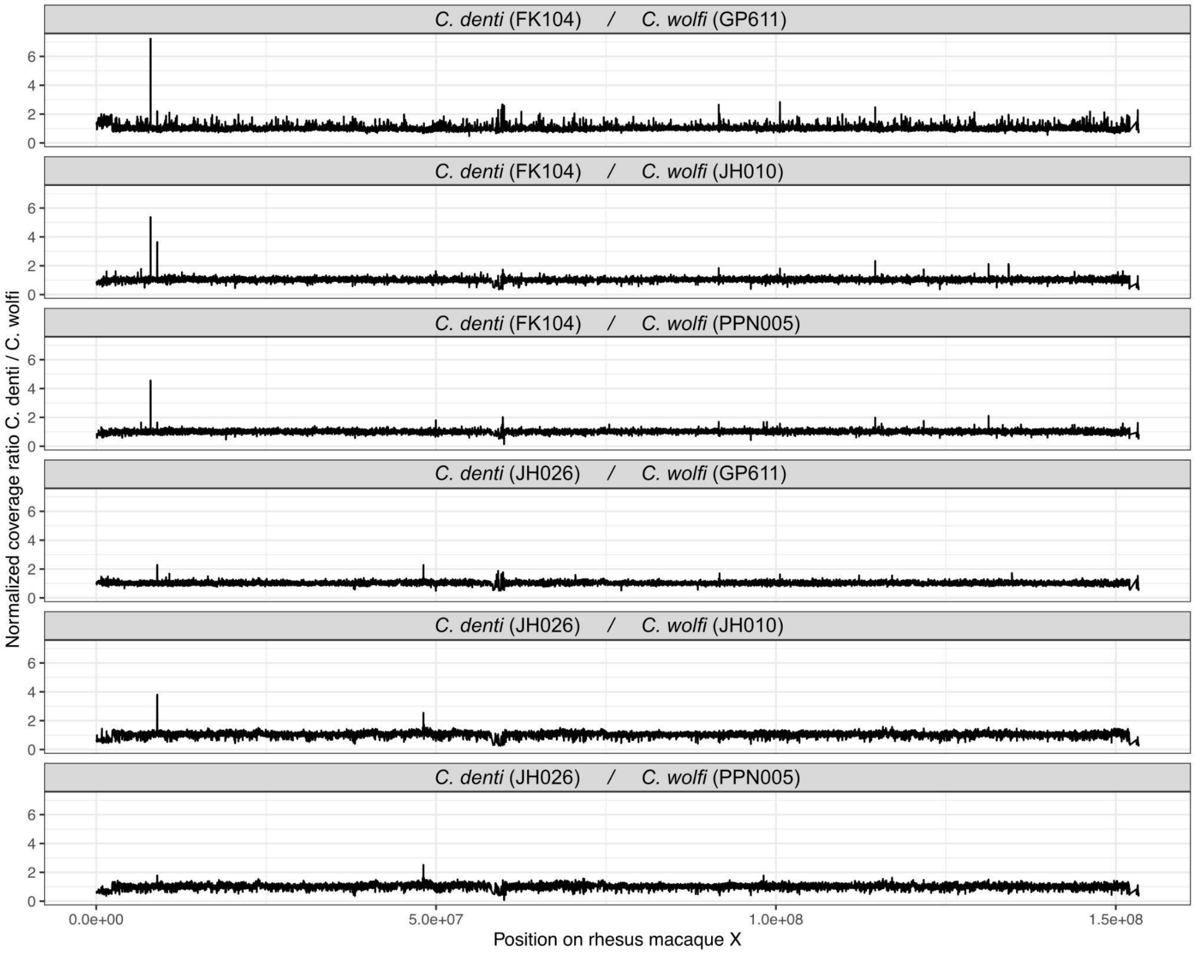

Supplementary Figure 14. Normalized coverage ratio between all combinations of *C. denti* and *C. wolfi* samples in windows along the rhesus macaque X chromosome. Coverage was normalized by dividing the mean coverage in each window with the X-chromosome wide average for each sample. Source data are provided as a Source Data file.

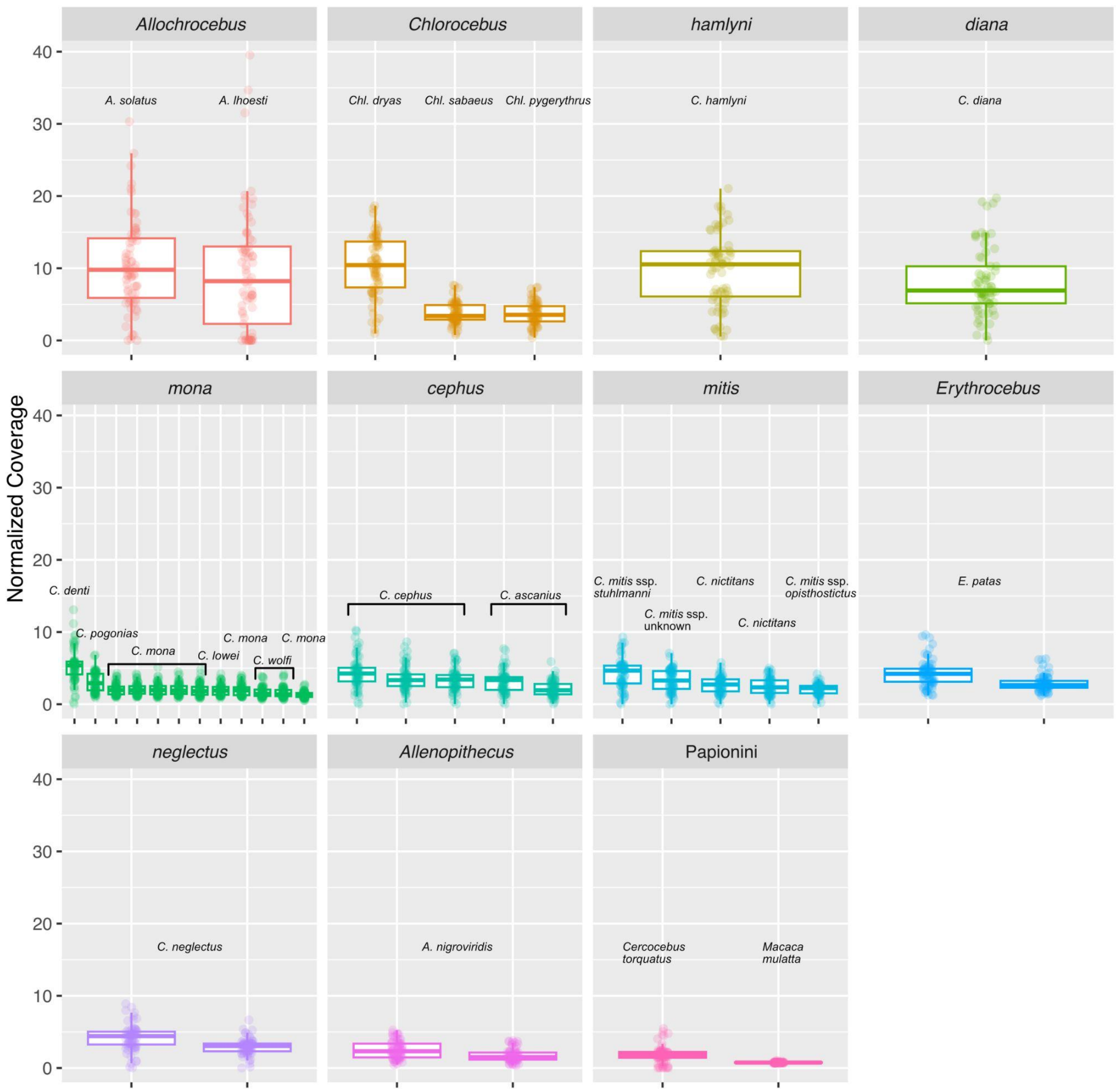

Supplementary Figure 15. Mapped read depth in the putative ampliconic region on the guenon Y-chromosome, normalized by the average Y-chromosomal coverage. Each point represents a non-overlapping 5 kb window in the rhesus macaque Y-chromosomal region 8,650,000-9,000,000 bp. Boxplot elements: center line, median; hinges, first/third quartile; whiskers, observations in 1.5x interquartile range. Source data are provided as a Source Data file.
