# Supplementary material for: Y chromosome introgression between deeply divergent primate species
Source: Nat Commun. 2024 Nov 29;15:10398. doi: 10.1038/s41467-024-54719-8 (PMC11607401; doi:10.1038/s41467-024-54719-8)
Supplement: Supplementary file 3 — Description of Additional Supplementary Files [file 41467_2024_54719_MOESM3_ESM.pdf]

The Supplementary Data file contains the Supplementary Data 1-5.

Supplementary Data 1. Sample information.

Supplementary Data 2. TSPY Alignment. Concatenated and trimmed alignment, constructed from two fragments of the Y-linked *TSPY* gene.

Supplementary Data 3. Autosomal D-statistics with *mitis* group taxa as P3, *C. denti* as P2 and all other *mona* group taxa as P1, using *Macaca mulatta* as outgroup. All sample combinations among these groups were tested. The p-values are included as estimated in Dsuite using the block-jackknife standard error, not corrected for multiple testing. We treated values of D with Z-scores > 3 as statistically significant.

Supplementary Data 4. Overview of annotated Y-chromosomal genes in guenons, and the prevalence of fixed amino acid substitutions between *C. denti*/*C. mitis* and *C. wolfi*/*C. pogonias*. Genes highlighted in green passed our filtering criteria and contain fixed amino acid substitutions. Genes highlighted in red failed the filtering criteria (see main text for details).

Supplementary Data 5. Summary of HyPhy meme test results for episodic diversifying selection.
