# Supplementary material for: Y chromosome introgression between deeply divergent primate species
Source: Nat Commun. 2024 Nov 29;15:10398. doi: 10.1038/s41467-024-54719-8 (PMC11607401; doi:10.1038/s41467-024-54719-8)
Supplement: Supplementary file 5 — Reporting Summary [file 41467_2024_54719_MOESM5_ESM.pdf]

nature portfolio

Corresponding author(s): Axel Jensen
Katerina Guschanski
Kate M. Detwiler

Last updated by author(s): Nov 10, 2024

Reporting Summary

Nature Portfolio wishes to improve the reproducibility of the work that we publish. This form provides structure for consistency and transparency in reporting. For further information on Nature Portfolio policies, see our Editorial Policies and the Editorial Policy Checklist.

Statistics

For all statistical analyses, confirm that the following items are present in the figure legend, table legend, main text, or Methods section.

n/a	Confirmed	
☐	☒	The exact sample size (*n*) for each experimental group/condition, given as a discrete number and unit of measurement
☒	☐	A statement on whether measurements were taken from distinct samples or whether the same sample was measured repeatedly
☐	☒	The statistical test(s) used AND whether they are one- or two-sided<br>*Only common tests should be described solely by name; describe more complex techniques in the Methods section.*
☒	☐	A description of all covariates tested
☐	☒	A description of any assumptions or corrections, such as tests of normality and adjustment for multiple comparisons
☐	☒	A full description of the statistical parameters including central tendency (e.g. means) or other basic estimates (e.g. regression coefficient) AND variation (e.g. standard deviation) or associated estimates of uncertainty (e.g. confidence intervals)
☐	☒	For null hypothesis testing, the test statistic (e.g. F, t, r) with confidence intervals, effect sizes, degrees of freedom and P value noted<br>*Give P values as exact values whenever suitable.*
☐	☒	For Bayesian analysis, information on the choice of priors and Markov chain Monte Carlo settings
☒	☐	For hierarchical and complex designs, identification of the appropriate level for tests and full reporting of outcomes
☒	☐	Estimates of effect sizes (e.g. Cohen's *d*, Pearson's *r*), indicating how they were calculated

Our web collection on statistics for biologists contains articles on many of the points above.

Software and code

Policy information about availability of computer code

Data collection	No software was used for data collection.
Data analysis	Software used to analyze the data in this project: Picard/2.23.4, bwa/0.7.17, QualiMap/2.2.1, GATK/4.2, samtools/1.19, ASTRAL/5.7.4, IQTREE/2.2.2.6, MitoFinder/1.4.1, TRIMMOMATIC/0.39, MAFFT/7.407, PAML/4.9j, tracer/1.7.1, Geneious R11 11.0.5, PhyML/3.3, ABBABABAwindows.py (https://github.com/simonhmartin/genomics_general), PopArt/1.7, Dsuite/0.4, Pixy/1.2.5, bpp/4.6.2, bppr/0.6.3, msprime/1.2, SLiM/4.0.1, HyPhy/2.5.51, R/2023.09.1+494, Python/3.7.4. Analytical scripts/workflows have been deposited to https://github.com/axeljen/denti_ychrom_scripts.

For manuscripts utilizing custom algorithms or software that are central to the research but not yet described in published literature, software must be made available to editors and reviewers. We strongly encourage code deposition in a community repository (e.g. GitHub). See the Nature Portfolio guidelines for submitting code & software for further information.

Data

Policy information about availability of data

All manuscripts must include a data availability statement. This statement should provide the following information, where applicable:

- Accession codes, unique identifiers, or web links for publicly available datasets
- A description of any restrictions on data availability
- For clinical datasets or third party data, please ensure that the statement adheres to our policy

Whole genome sequencing data generated for this project are available at ENA under accession number PRJEB73870 (https://www.ebi.ac.uk/ena/browser/view/PRJEB73870), with individual accession codes listed in Supplementary Data 1. Previously published whole-genome sequencing data used in this project is accessible at SRA under project accession numbers PRJEB67744 [https://www.ncbi.nlm.nih.gov/bioproject/1031930], PRJEB32105 [https://www.ncbi.nlm.nih.gov/bioproject/572824], PRJNA240242 [https://www.ncbi.nlm.nih.gov/bioproject/240242], PRJNA251548 [https://www.ncbi.nlm.nih.gov/bioproject/251548], PRJNA595456 [https://www.ncbi.nlm.nih.gov/bioproject/PRJNA595456], and PRJNA512907 [https://www.ncbi.nlm.nih.gov/bioproject/512907], with sample specific accession codes listed in Supplementary Data 1. The TSPY sequence data generated for Supplementary Figure 5 is available as a Source Data file, and at GenBank under accession codes PQ570681, PQ570682, PQ570683, PQ570684, PQ570685, PQ570686, PQ570687, PQ570688 (Supplementary Data 2). Previously published TSPY used in Supplementary Figure 5 is available through accession codes EF517803.1 [https://www.ncbi.nlm.nih.gov/nuccore/EF517803.1/], AY450876.1 [https://www.ncbi.nlm.nih.gov/nuccore/AY450876.1/], AY048057.1 [https://www.ncbi.nlm.nih.gov/nuccore/AY048057.1], AY450874.1 [https://www.ncbi.nlm.nih.gov/nuccore/AY450874.1], EF517804.1 [https://www.ncbi.nlm.nih.gov/nuccore/EF517804.1], AY897616.1 [https://www.ncbi.nlm.nih.gov/nuccore/AY897616.1], AY665648.1 [https://www.ncbi.nlm.nih.gov/nuccore/AY665648.1], AY450878.1 [https://www.ncbi.nlm.nih.gov/nuccore/AY450878.1], AY048058.1 [https://www.ncbi.nlm.nih.gov/nuccore/AY048058.1], AY450877.1 [https://www.ncbi.nlm.nih.gov/nuccore/AY450877.1], EF517805.1 [https://www.ncbi.nlm.nih.gov/nuccore/EF517805.1], AY048059.1 [https://www.ncbi.nlm.nih.gov/nuccore/AY048059.1], AF284281.2 [https://www.ncbi.nlm.nih.gov/nuccore/AF284281.2], AY450880.1 [https://www.ncbi.nlm.nih.gov/nuccore/AY450880.1], AY450875.1, [https://www.ncbi.nlm.nih.gov/nuccore/AY450875.1], JN106053.1 [https://www.ncbi.nlm.nih.gov/nuccore/JN106053.1], JN106052.1 [https://www.ncbi.nlm.nih.gov/nuccore/JN106052.1], and EF517806.1 [https://www.ncbi.nlm.nih.gov/nuccore/EF517806.1]. The M. mulatta reference genome is available under the NCBI RefSeq accession number GCF_003339765.1 [https://www.ncbi.nlm.nih.gov/datasets/genome/GCF_003339765.1/]. Source data are provided as a Source Data file.

Research involving human participants, their data, or biological material

Policy information about studies with human participants or human data. See also policy information about sex, gender (identity/presentation), and sexual orientation and race, ethnicity and racism.

Reporting on sex and gender	n/a
Reporting on race, ethnicity, or other socially relevant groupings	n/a
Population characteristics	n/a
Recruitment	n/a
Ethics oversight	n/a

Note that full information on the approval of the study protocol must also be provided in the manuscript.

Field-specific reporting

Please select the one below that is the best fit for your research. If you are not sure, read the appropriate sections before making your selection.

☐ Life sciences ☐ Behavioural & social sciences ☒ Ecological, evolutionary & environmental sciences

For a reference copy of the document with all sections, see nature.com/documents/nr-reporting-summary-flat.pdf

Ecological, evolutionary & environmental sciences study design

All studies must disclose on these points even when the disclosure is negative.

Study description	We generated whole genome sequencing data from seven individuals from five primate species, and combined this with publicly available genomes to perform a comprehensive phylogenomic study of guenons (Cercopithecini) focusing on Y chromosome evolution. We also amplified and sequenced Y chromosome-linked markers from four additional Cercopithecus denti males.
Research sample	We combined all available high coverage genome data available from guenon taxa, and augmented this with newly generated data from several previously unsequenced taxa (Cercopithecus denti, C. wolfi elegans, C. wolfi wolfi, C. mitis stuhlmanni). Since our main focus was Y chromosome evolution, we aimed at maximizing male representation wherever possible, including novel sequencing of C. hamlyni (only a female sample was sequenced previously from this species). We also amplified and sequenced Y chromosome-

	linked markers from four additional Cercopithecus denti males, and combined this with publicly available guenon sequences of the same markers.
Sampling strategy	Samples were opportunistically collected from dead animals, or from feaces. We aimed at maximizing taxonomic representation of the guenons, aiming at having male representatives and prefereably more than one sample for as many species as possible.
Data collection	Samples for DNA extraction were opportunistically collected from dead specimen, or noninvasively from feaces. Sampling was led by K.M.D., J.A. and J.A.H., and details about sampled individuals (species, date, geographic location) was documented in field notebooks (pen and paper) and transcribed into digital (Excel) data sheets.
Timing and spatial scale	Samples collected for this study were aquired between 2005-2016 in Democratic Republic of the Congo and Rwanda.
Data exclusions	No data was excluded.
Reproducibility	Lab protocols, sequencing techniques and analytical approaches are described in the methods. The raw sequencing data is deposited to European Nucleotide Archive, and analytical code used in the project is available on github.
Randomization	N/a
Blinding	N/a

Did the study involve field work? ☒ Yes ☐ No

Field work, collection and transport

Field conditions	Samples were opportunistically collected from deceased specimen in Democratic Republic of the Congo across 8 years (2008-2016). One fecal sample was collected in Rwanda in 2005.
Location	The samples were aquired from different locations in Democratic Republic of the Congo and Rwanda, latitude and longitude data for the collected samples are listed in the supplementary table 1.
Access & import/export	Samples were aquired and exported in compliance with relevant legislation. Export permits were aquired from local and international organizations as follows:<br>Cercopithecus denti (field ID M82/NF03, used for Y chrom marker sequencing): ORTPN & PCFN Export Permit, Nyungwe National Park Authority in Rwanda<br>C. denti (FK103, Y chrom markers): USFWS Cleared 10/20/2008; ICCN/MB/DT/DG/2008-01188,<br>C. denti (JH25, Y chrom markers): USFWS Cleared 06/28/2016; CITES PERMIS No. 6718,<br>C. denti (ME402, Y chrom markers): USFWS Cleared 10/20/2008; ICCN/MB/DT/DG/2008-01188,<br>C. denti (FK104, whole genome sequencing [WGS]): USFWS Cleared 10/20/2008; ICCN/MB/DT/DG/2008-01188,<br>C. denti (JH026, WGS): USFWS Cleared 06/28/2016; CITES PERMIS No. 6718,<br>C. wolfi elegans (JH010, WGS): USFWS Cleared 08/22/2009 ; ICCN/DG/ADG/KBY/2009-0660 (CITES 3245),<br>C. wolfi elegans (GP611, WGS): USFWS Cleared 08/22/2009 ; ICCN/DG/ADG/KBY/2009-0660 (CITES 3245),<br>C. wolfi wolfi (PPN005, WGS): USFWS Cleared 06/28/2016; CITES PERMIS No. 6719,<br>C. hamlyni (GP612, WGS): USFWS Cleared 10/20/2008; ICCN/MB/DT/DG/2008-01188,<br>C. mitis stuhlmanni (ME403): USFWS Cleared 10/20/2008; ICCN/MB/DT/DG/2008-01188
Disturbance	No disturbance, since we opportunistically collected the samples from dead animals or noninvasively from feaces on the ground.

Reporting for specific materials, systems and methods

We require information from authors about some types of materials, experimental systems and methods used in many studies. Here, indicate whether each material, system or method listed is relevant to your study. If you are not sure if a list item applies to your research, read the appropriate section before selecting a response.

Materials & experimental systems

n/a	Involved in the study	
☒	☐	Antibodies
☒	☐	Eukaryotic cell lines
☒	☐	Palaeontology and archaeology
☐	☒	Animals and other organisms
☒	☐	Clinical data
☒	☐	Dual use research of concern
☒	☐	Plants

Methods

n/a	Involved in the study	
☒	☐	ChIP-seq
☒	☐	Flow cytometry
☒	☐	MRI-based neuroimaging

Animals and other research organisms

Policy information about studies involving animals; ARRIVE guidelines recommended for reporting animal research, and Sex and Gender in Research

Laboratory animals	No laboratory animals were used in this study.
Wild animals	We opportunistically collected tissue samples from dead animals, or took a noninvasive fecal sample, as follows:<br>Cercopithecus denti (field ID M82/NF03, used for Y chrom marker sequencing, Rwanda): Fecal sample from a male individual, age unknown.<br>C. denti (FK103, Y chrom markers, DRC): Ear snip from dead male individual, age unknown.<br>C. denti (JH025, Y chrom markers, DRC): Unknown organ tissue from a dead male, age unknown.<br>C. denti (ME402, Y chrom markers, DRC): Ear snip from dead male, age unknown.<br>C. denti (FK104, whole genome sequencing [WGS], DRC): Ear snip from dead male, age unknown.<br>C. denti (JH026, WGS, DRC): Unknown organ tissue from a dead female, age unknown.<br>C. wolfi elegans (JH010, WGS, DRC): Ear snip from dead male, age unknown.<br>C. wolfi elegans (GP611, WGS, DRC): Ear snip from dead female, age unknown.<br>C. wolfi wolfi (PPN005, WGS, DRC): Kidney sample from dead feamle, age unknown.<br>C. hamlyni (GP612, WGS, DRC): Ear snip from dead male, age unknown.<br>C. mitis stuhlmanni (ME403, DRC): Liver sample from dead male, age unknown.
Reporting on sex	We focus on Y chromosome evoution in this project, which is a male-restricted chromosome. Sex was determined molecularly/bioinformatically, either by amplification of Y chromosome specific markers or by sequencing coverage of the sex chromosomes.
Field-collected samples	Samples were stored in RNAlater or 94% ethanol.
Ethics oversight	Field work and sample collection was performed under oversight of TL2 Project, Lukuru Wildlife Research Foundation, Congolese Institute for Nature Conservation (ICCN), and the Rwandan Development Board (formerly ORTPN and PCFN) in compliance with local and international legislation.

Note that full information on the approval of the study protocol must also be provided in the manuscript.

Plants

Seed stocks	*Report on the source of all seed stocks or other plant material used. If applicable, state the seed stock centre and catalogue number. If plant specimens were collected from the field, describe the collection location, date and sampling procedures.*
Novel plant genotypes	*Describe the methods by which all novel plant genotypes were produced. This includes those generated by transgenic approaches, gene editing, chemical/radiation-based mutagenesis and hybridization. For transgenic lines, describe the transformation method, the number of independent lines analyzed and the generation upon which experiments were performed. For gene-edited lines, describe the editor used, the endogenous sequence targeted for editing, the targeting guide RNA sequence (if applicable) and how the editor was applied.*
Authentication	*Describe any authentication procedures for each seed stock used or novel genotype generated. Describe any experiments used to assess the effect of a mutation and, where applicable, how potential secondary effects (e.g. second site T-DNA insertions, mosiacism, off-target gene editing) were examined.*
